# The important role of community organizations in stroke recovery and reintegration

**DOI:** 10.3389/fstro.2024.1430935

**Published:** 2024-07-25

**Authors:** Michelle L. A. Nelson, Evan MacEachern, Marianne Saragosa

**Affiliations:** ^1^Science of Care Institute, Lunenfeld-Tanenbaum Research Institute, Toronto, ON, Canada; ^2^Sinai Health, Toronto, ON, Canada; ^3^Institute of Health Policy, Management and Evaluation, University of Toronto, Toronto, ON, Canada

**Keywords:** stroke, rehabilitation, community, discharge, transitions, voluntary sector

## Abstract

Stroke systems of care are grappling with pressures to ensure high-quality, evidence-informed, person-centered services with an emphasis on safe and timely discharge to the person's home and community. The literature describing the need for robust stroke systems has focused on services within the healthcare system, which are predominantly hospital-based and guided by the Medical Model. However, given the long-term nature of stroke recovery and the importance of attending to the challenges of resuming a meaningful life post-stroke, the involvement of community organizations becomes paramount in providing longer term support. This perspective paper explores the engagement of “community” within the context of stroke systems of care. It proposes that the community is both a destination of the pathway and a partner that can help address the ongoing and often unmet needs experienced post-discharge. Through these partnerships and collaborations, we suggest that community organizations can fill service gaps; volunteers could be leveraged to expand the breadth and quality of health and social services to meet the needs of stroke survivors and their families.

## Introduction

Despite highly developed care programs with substantial evidence and strong advocacy, stroke survivors face significant health and social challenges after transitioning into the community, where hospital readmissions are common (Olson et al., 2013). Fragmentation during care transitions can lead to adverse outcomes, including readmissions and decreased patient satisfaction. When transitioning home after a stroke, the involvement of community organizations is crucial for providing long-term support (Wolfe et al., 2000; Attard et al., 2020; Norlander et al., 2022; Lo and Chau, 2023). Engaging community organizations has been proposed as a key approach for addressing these unmet needs.

Stroke systems of care are under pressure to ensure high-quality, evidence-based, and person-centered services and emphasize safe and timely discharge to home and community. These priorities, particularly those related to transitions of care, raise questions about the role and contributions of the broader community in supporting stroke survivors. Partnerships between health services and the voluntary sector can offer innovative solutions to improve patient care and address the multifaceted needs of individuals post-discharge. We suggest that “community” within stroke systems of care can be viewed both as a destination at the end of health service pathways and as a partner in addressing the ongoing, often unmet needs post-discharge. This is well aligned with the World Health Organization's definition of person-centered care, which emphasizes placing people and their communities at the center of all service design and delivery:

“People-centered care extends the concept of patient-centered care to individuals, families, communities, and society. On the other hand, patient-centered care focuses on the individual seeking care. Patient—people-centered care encompasses these clinical encounters and includes attention to the health of people in their communities and their crucial role in shaping health policy and health services.”

Given the increasing attention to civil society and community organizations, often referred to as “third-sector organizations” (TSOs), it is imperative to critically consider their current engagement in stroke care transitions.

## Engaging the community within resource-constrained healthcare systems

With increasing patient complexity, new reimbursement models, and pressures to reduce costs while improving care quality and patient experiences, it would be short-sighted to underestimate the potential impact of the voluntary and community sectors within healthcare and support systems. When combined with decreasing compensation for readmissions, Robert Waller, former CEO of Mayo Clinic, noted that “the United States has three options: (i) it can spend more on healthcare—which hardly seems possible, (ii) it can help less—which is unconscionable, or (iii) it can redesign healthcare by turning to the power of community to redefine healthcare and pursue true health” (Waller, 2012). During a meeting of 43 leading U.S. healthcare organizations, the Health Systems Learning Group acknowledged that as hospitals and health systems struggle with challenges such as uncompensated care, emergency department overuse, and readmissions, the need for transformative community partnerships becomes increasingly clear. The CEO of the Henry Ford system, Nancy Schlichting, remarked, “We're changing the center of gravity from the hospital to the home and the community” [Health Systems Learning Group (HSLG), 2013]. While such statements champion advocacy, they lack operational clarity on who represents the community. Are we referring to the broader civil society or the formal organizations that provide civic engagement and societal wellbeing?

Through partnerships and collaborations, TSOs can cost-effectively “fill gaps;” volunteers can expand the breadth and quality of health and social services (Brinkerhoff and Brinkerhoff, 2011; Hushie, 2016). Most communities have a long history of volunteerism and civic engagement. These services traditionally compensated for social support gaps and fostered meaningful community life (Elson, 2009). Civil society organizations are vital sources of health-promoting capabilities, bringing people together around common causes and promoting resilience and wellbeing through social connections. Associational life has been recognized as significant, with social and communal activities positively impacting physical, mental, and emotional wellbeing (Lindsay-Smith et al., 2019). Community initiatives extend beyond institutional-based practices to benefit spirituality, culture, and mindfulness.

TSOs address many social determinants of health by providing services close to where people live and acquiring a deep understanding of the community's need for innovation. Such understanding of their members is facilitated by drawing trusted staff and volunteers from the community they serve (Buckingham, 2009; Dickinson et al., 2012). TSOs embedded within geographic and culture-sharing communities can support “hardly reached” groups (Wilson et al., 2012), navigating cultural, political, and psychosocial considerations in program implementation. A key contribution of the voluntary and community sector within stroke pathways is their flexibility; they are malleable and not solely directed by public health mandates or funding requirements. TSOs may develop timely interventions based on emerging insights, conduct rapid evaluations, and determine the feasibility of scaling interventions before academic studies are completed.

## Positioning TSOs as partners in the stroke pathway

To support the broader engagement of TSOs in stroke care, we propose three key changes: first, challenge the medical hegemonic nature of stroke care; second, recognize that stroke recovery extends beyond hospital-based care and that the post-discharge period is critical, often outside the timelines and reach of healthcare services; and, third, generate rigorous evidence regarding the effectiveness and value of TSOs' work.

Literature on robust stroke systems focuses on health services, predominantly hospital-based and guided by the medical model (Gannon, 2023), which seeks to prevent, manage, or cure disease using evidence-based medicine (Fuller, 2017). Authors advocating for improved stroke services often highlight policy changes but rarely acknowledge community organizations' role in lobbying policymakers. This is problematic because health is derived, in part, from social conditions, and medical interventions cannot address the root causes of poor transitional experiences, such as inappropriate housing, a lack of transportation, food insecurity, or caregiver strain. Addressing the root causes of these social issues is essential, working with citizens' strengths and community assets. There is substantial evidence that TSOs enhance health systems through advocacy and research (Blas, 2008). TSOs' deep community connections provide platforms for citizens to influence health and social care development by engaging patients and families in service planning essential for person-centered care (Sanders et al., 2004; Bull et al., 2024). TSOs can influence policy and clinical practice by deploying best-practice recommendations in stroke care.

Approximately 50% of stroke survivors live with a disability that affects their independence in daily tasks (Kong and Lee, 2014). While stroke recovery gains are greatest during the period of hospital admission to discharge (Horgan et al., 2009; Kong and Lee, 2014), evidence indicates that people may still improve in both activities of daily living and instrumental activities of daily living past the rehabilitation phase (Demain et al., 2006; Horgan et al., 2009; Kong and Lee, 2014; Bernhardt et al., 2017; Engel-Yeger et al., 2018; Ballester et al., 2019), which is typically outside the average hospital stay (Chien et al., 2020; Bijl et al., 2023; Tran et al., 2023). This underscores the importance of community-based stroke services, which can be vital in long-term recovery and psychosocial support. Furthermore, systematic evidence from 19 surveys showed a high prevalence of long-term unmet needs among stroke survivors, with a median of 2–5 unmet needs per person, including access to information, transportation, home help, personal care, and ongoing therapy (Chen et al., 2019). The highest prevalence of unmet needs was observed at 6 months (62%) and 2 years (81%) post-stroke (Lin et al., 2022). Given the pressures on health systems, hospital-based recovery and rehabilitation cannot address all these unmet needs over such a long timeframe.

Unfortunately, the contributions of community partners in improving patient experience and system efficiency are often under-recognized despite their widespread involvement in stroke recovery services. Many clinicians refer patients to these programs for peer support, education, self-management training, befriending, and aphasia programs. Despite TSO engagement, research on transitional support focuses on services delivered by healthcare professionals. However, services provided by lay navigators or volunteers are noted to support transitions from hospital to home successfully (Egan et al., 2010; Lorhan, 2013). Evidence regarding community-level engagement in health promotion, client recovery, and service delivery strongly suggests that challenges in supporting hardly reached populations lie in developing culturally relevant interventions and understanding the patient population holistically—opportunities that community organizations can address. Recognizing the value of services offered by TSOs, thought leaders at the King's Fund (Imison and Bohmer, 2013), the American Hospital Association, and the Beryl Institute (Garrison and Wolf, 2016) have long advocated for the more purposeful engagement of TSOs in healthcare organization and delivery. To achieve system-level integration of TSOs, research on facilitators and barriers to engagement at organizational and system levels is required.

## Conclusion

Future healthcare must integrate clinical and social interventions tailored to respond holistically to people's health needs. Partnerships with community organizations are essential to robust systems of care, offering longevity of services, proximity to service users, and sensitivity to their diverse needs.

## Data availability statement

The original contributions presented in the study are included in the article/supplementary material, further inquiries can be directed to the corresponding author.

## Author contributions

MN: Conceptualization, Resources, Supervision, Writing – original draft, Writing – review & editing. EM: Writing – original draft, Writing – review & editing. MS: Writing – original draft, Writing – review & editing.
